# Science may be objective, scientists are not always

**DOI:** 10.52054/FVVO.13.1.012

**Published:** 2021-03-31

**Authors:** Becker S

**Affiliations:** Sven Becker, MD, PhD, Director, Frankfurt University Women’s Hospital, Goethe-University, Frankfurt, Germany.

“Historically, most things, most people ever believed to be true eventually turned out not to be true.“

We live in the age of science. At least, that is what we like to believe. With the science-driven industrial revolution starting in the 18^th^ century, and the science-driven revolution of academic medicine starting in the second half of the 19^th^ century, we have become accustomed to knowing more and more about this world in an objective and scientific way. And, curiously, every generation naturally believes, they know everything.

Looking back, it is hard to understand, how our forefathers could have believed in bloodletting as the major medical intervention for centuries. It was seen as “the truth“ and people who might have doubted its rationale were marginalised. Today we know it was misguided at best and probably killed innumerably more patients than it saved. Then there was the world before we understood the true role of bacteria. It is curious to read about the treatment of tuberculosis before the invention of antibiotics. In Thomas Mann’s Zauberberg (The Magic Mountain), we encounter a world of scientifically based treatments, that today leave us bewildered. Reading Albert Camus’s “La Peste” leaves us wondering: why don’t they just take the antibiotics and get it over with.

What was the best available “scientific” evidence in the past, is often a curiosity today. With this in mind, we should take a critical look at current scientific facts and controversies and we have good reason to do so.

Truth, fake news and alternative facts have kept us busy in the world of politics; we all know that. It is a naive thought to believe that the world of science would be immune to these problems. In its review of the most read articles of 2020, JAMA, the Journal of the American Medical Associations, allows some insight into the politics of science and the power of scientific fashions. On January 15 th 2020, Rita Rubin published a commentary entitled “Backlash Over Meat Dietary Recommendations Raises Questions About Corporate Ties to Nutrition Scientists“ (Rubin 2020).

Ms. Rubin described the reaction from certain quarters to publications questioning the widely accepted dangers of red meat consumption. Apparently, red meat consumption is not as unhealthy as often portrayed or, more precisely, the scientific base for such claims is very small. She wrote “Annals Editor-in-Chief Christine Laine, MD, MPH, saw her inbox flooded with roughly 2000 emails—most bore the same message, apparently generated by a bot—in a half hour. Laine’s inbox had to be shut down, she said. Not only was the volume unprecedented in her decade at the helm of the respected journal, the tone of the emails was particularly caustic.“ These were emails criticising the planned publication.

It is important to reflect on what is going on here. This was BEFORE the article was actually published, i.e. an effort to suppress its publication after it had gone through a peer review process with someone from that peer-review process leaking the information to interested (and apparently highly opinionated) circles. Of course, the criticism was not: “I don’t like the article, because it goes against what I think“, it was attacking the scientific base suggesting “This article is not scientific and thus must not be published“.

Reproductive endocrinology colleagues report similar behind-the-scenes interactions prior to the recent metanalysis about hormone replacement therapy (HRT) which put the breast cancer risk of HRT in an in- teresting perspective (Vinogradova et al., 2020). After the WHI-study from 2002 which widely discredited HRT was shown more than 10 years later to have been severely mispresented at the time, the ideological nature of a fight about very small risks has become obvious. Roger Lobo provides an interesting review and commentary in Nature Review Endocrinology in 2017. He particularly comments on how the data was presented by NIH officials without input from the original researchers (Lobo, 2017).

HRT, meat consumption are topics that are being discussed within a legal, economic and ideological context. Science does have a powerful objective aspect; however, it is subject to the same passions and manipulations as all political discourse. Inevitably, these controversies always surround what I call “Issues of small risk“, when small but discernible risks, that are statistically present, are unclear and thus disputed with regard to their clinical relevance.

In our field of surgical gynaecology we should be careful to identify our own “ideological” disputes and review the data accordingly. Pretending to “let the science speak for itself“ is a typical phrase used by those who are already misrepresenting it. Science does not speak: scientists do. And scientists are human. They have agendas, biases and skeletons in the closet, too.

The role of morcellation is such a topic, heavily influenced by the legal system in the United States. Scientific facts suggesting very small risks were surrounded and masked by an emotive issue and a devastating diagnosis. While the FDA placed the risk of the occult leiomyosarcoma at 1/400, a recent Cochrane review puts it at 1/2000 (Zullo et al., 2020). It is always worthwhile to also recall the controversy surrounding silicone breast implants and their alleged effect on autoimmune disease in the 90’s. After 5 Billion US-Dollars had been dispersed in legal fees, the ultimate conclusion, 15 years after the allegation, was that there is no connection.

The controversy surrounding minimally invasive surgery and cervical cancer is another subject. Here too, small risks are demonstrated once and then carried around as immutable facts, much like the monstrance during the catholic processions. And beyond our own specialty, the discussions surrounding the current coronavirus pandemic and the ongoing controversies about climate change are battles about who owns the truth. The answer must be: probably no-one does.

Science is always a clash of opinions and opinions are held with passion. While science can offer glimpses of objectivity, we must never forget: it is a tool in the hands of passionate women and men who, much as they strive, can never be completely objective. As long as we remember that, science will continue to move ahead. Hesitantly, erringly on a path which, maybe, will lead us to the Truth one day.
